# 

**Published:** 1893-12

**Authors:** 


### LITERARY.

With the December number MCCLURE'S MAGAZINE begins its second volume, and, by the character of its contents, renews the promise, so amply kept in the first volume, of being a magazine, not to buy and lay by for a day of future reference that never comes, but to buy and read—read at once, read without stopping. An account by Mr. Arthur Warren of a visit to Archdeacon Farrar, embracing much interesting talk by the Archdeacon on his acquaintance with Tennyson and other eminent men, on his work, and on Westminster Abbey, and richly illustrated, opens the number. Professor Henry Drummond relates the history of the founding and progress of the Boys' Brigade, an organization which is making a new earth for the street Arab and other untended, or half-tended boys. The article of sharpest present interest, perhaps, is a discriminating and sympathetic study of the character and career of Governor William McKinley, by E. Jay Edwards. Charles A. Dana, the distinguished editor of the "New York Sun," provides a particularly interesting travel sketch in some notes on a journey he lately made to Jerusalem: Nearly every article is copiously illustrated, but of a special value are a group of portraits of Tennyson and his friends—among them Sir John Herschel, Browning, Charles Darwin and Carlyle—reproduced from the famous negatives of Mrs. Julia Margaret Cameron. The last of he Sherlock Holmes stories by Conan Doyle, an Arkansas Christmas story by Octave Thanet, and a new story of the seen and unseen by Mrs. Oliphant, give special distinction to the fiction of the number. In the novel department of "Human Documents," portraits are given of the Honorable Whitelaw Reid, William T. Stead, and Governor William McKinley, from boyhood to the present time.

A Japanese pupil of Mr. Lafcadio Hearn has asked him in horror and amazement how it is that the strange subjects of love and marriage are so freely treated in English novels. This gives Mr. Hearn occasion to tell in his article, "Of the Eternal Feminine," in the December ATLANTIC, how different a place women occupy in Japan and in America or Europe. Equally noteworthy is Mr. F. B. Sanborn's article on "Thoreau and his English Friend Thomas Cholmondeley." The paper is made up mainly of letters between a young Englishman of no common character and the naturalist and philosopher whose name is coming more and more to be coupled, like Emerson's and Hawthorne's, with Concord in its best days. Mrs. Wiggin provides the short story of the number in "Tom o' the Blueb'ry Plains," a pathetic sketch of New England life. Mrs. Cavazza's story, "The Man from Aidone," has its third, last, and most effective part. Charles Egbert Craddock continues "Vanished Star." Studies of nature are nearly always expected in THE ATLANTIC, and from Mr. Bradford Torrey and Mr. Frank Bolles the readers of the magazine have learned to expect very charming papers. Such, indeed, are "In the Flat Woods," by Mr. Torrey, and "Birds at Yule-Tide." by Mr. Bolles. To these are added the vivid pictures of Mr. Hamlin Garland's "Western Landscapes." An unsigned paper, "Ideal Transit," suggests, half whimsically, a pleasant solution of all the difficulties of travel. Professor Woodrow Wilson, in "Mere Literature," makes a plea for the study of books not as subjects of scientific inquiry. "Democracy in America," by Professor Francis Newton Thorpe, is of interest particularly to students of our social history. "The Blazing Heart," a poem by Mrs. Alice Williams Brotherton, and the usual departments, fill out this strong concluding number of THE ATLANTIC'S one hundred and thirty-fourth volume.—Houghton, Mifflin & Co., Boston.

The illustrated articles are an important feature in the December POPULAR SCIENCE MONTHLY. The number opens with an account by President Jordan, of Stanford University, of the behavior of a South Sea monkey in the various surroundings of human civilization. It is called the "Story of Bob," and is a delightful mixture of scientific observation and comical incident. Several of Bob's most interesting feats are shown in pictures. The "Modern War Vessels of the United States Navy" are described by W. A. Dobson, their means of defense and offense being fully explained. The article is illustrated with views of the cruiser New York, the monitor Miantonomoh, and other typical vessels. Another copiously illustrated article is "The Fruit Industry in California," by Charles Howard Shinn, the pictures comprising views of orchards, specimen trees and branches of fruit. Prof. G. H. Perkins contributes a paper on "The Calumet in the Champlain Valley," in which thirteen forms of Indian pipes are figured. Prof. Huxley's Romanes lecture on "Evolution and Ethics" is concluded in this number, and is followed by a critical letter from Robert Mathews. This lecture also furnishes Leslie Stephen, with a text for a discussion of "Ethics and the Struggle for Existence." Prof. Warren Upham tells what answers are given to the question, "How Old is the Earth?" Miss Abby L. Alger contributes a myth of "The Creation," told to her by a Penobscot Indian. The results of some of Lombroso's recent researches upon "Criminal Woman," are set forth by Miss Helen Zimmern. "Sir Daniel Wilson" is the subject of the usual "Portrait and Sketch," the latter being furnished by Horatio Hale.

Volume I. of the two-volume edition of the Funk & Wagnalls Standard Dictionary of the English language, will be issued on December 16th. This volume has been four years in making; two hundred and thirty-eight editors and specialists have been employed upon it; and the cash outlay has been about a half million dollars. The advance orders for the work mount up into the tens of thousands.

The following letter was received by the publishers from a well known gentleman prominently identified with the late World's Fair at Chicago:

*Mines and Mining Building, Jackson Park, Ill.* Messrs. FUNK & WAGNALLS:

*Gentlemen*: I am pleased to inform you that the Standard Dictionary has been granted an award (diploma and medal) in group No. 150. The exact wording of all the awards will not be announced for probably three or four weeks.

The vocabulary of the Standard Dictionary is extraordinarily rich and full, that of no other dictionary nearly equaling it, although great care was taken to throw out all useless words.

The complete novel in the December number of LIPPINCOTT'S is "Sergeant Crœsus," by Captain Charles King. It is one of his most interesting tales of army life and Indian fighting in the wild West, and makes a new departure in having a private and a foreigner for its hero.

The tenth and last of Lippincott's Notable Stories, "When Hester Came," will be found to be one of the very best, as it is the longest, of the series. It is by an entirely new and very promising writer, Mrs. Bride Neill Taylor, of Texas.

Another story of marked power, at once striking delicate and pathetic, is "In the Camp of Philistia," by Virginia Woodward Cloud. "A Dream in the Morning," by Alice Brown, is a brief and beautiful sketch of a soul's undying devotion in the future life.

The Journalist Series is continued in "A Newspaper Sensation," by Louis N. Megargee, who tells of "a clever capture" which greatly discouraged grave robbing in a certain region. The facts will be remembered by many.

The World's Fair will not be permitted to live only in the memories of those who saw it, and in the files of newspapers. The Bancroft Company, Auditorium Building, Chicago, have in preparation what they call THE BOOK OF THE FAIR, which will be a permanent and illustrated chronicle of the exhibits. The text is by Hubert Howe Bancroft, and the illustrations profuse. As pointed out in the preface, the exhibition of 1851 was contained in a single edifice of one million square feet, while the space occupied at the World's Fair of to-day is eight or ten times as great.

Those who desire to engage in the lucrative occupation of canvassing for the Book of the Fair should communicate with Rhule, Thomas & Co., 24 Park Place, New York city.

The multiplicity and excellence of other magazines, far from lessening the usefulness of the REVIEW OF REVIEWS, makes this unique periodical more and more a necessity. Its indexes, condensations of leading articles, classified lists of new books, and general survey of things, written things said, and things done during the month preceding its issue, would suffice to keep the busy reader in touch with the current of life and thought, even if he were able to read nothing else. The December number is as full of variety and freshness as its predecessors have regularly been ; and to those who know the REVIEW OF REVIEWS this is a sufficient commendation.

THE HOME JOURNAL is the exponent of that literary and art culture which gives grace and refinement to social intercourse—a society journal in the best sense of the term. Out-of-town readers will find the best life of the metropolis reflected in its pages. It is an international journal and by its foreign correspondence and essays brings its readers *en rapport* with the social life of the great European centres. The HOME JOURNAL addresses its editorial and advertising columns to people of culture and fashion. It is essentially a paper for the home, a home journal, and the only HOME JOURNAL.

A Merry Christmas for you and yours, will be greatly enhanced if you get a copy of the December issue of TABLE TALK. It is full of suggestions to help you in your preparations for this holiday season, in the way of menus, recipes, all foods and decorations, popular at this time. Be sure you see this number. It is published by Table Talk Publishing Co., Philadelphia, at $1.00 a year or 10 cents a copy.

